# Unpacking gender discrepancies in academic promotion across STEM fields in Mexico

**DOI:** 10.1371/journal.pone.0324464

**Published:** 2025-08-14

**Authors:** Lauren Lanahan, Claudia Gonzalez-Brambila, Daniel Erian Armanios

**Affiliations:** 1 Lundquist College of Business, University of Oregon, Eugene, Oregon, United States of America; 2 Department of Business Administration, Instituto Tecnologico Autonomo de Mexico (ITAM), Mexico City, Mexico; 3 Saïd Business School, University of Oxford, Oxford, United Kingdom; Mansoura University Faculty of Veterinary Medicine, EGYPT

## Abstract

Gender inequality in the sciences remains a persistent issue. Women are often unable to participate in the scientific process as easily as men. When they do, this is largely constricted to opportunities at the lower rather than higher ranks of academia. This gap not only sets back female scientists but also scientific and social progress, more generally. The objective of this study is to look at a prominent national program for researchers in Mexico – Sistema Nacional de Investigadores (SNI) – to assess additional productivity and promotion heterogeneity by gender across career trajectories and disciplinary boundaries. Tracing productivity and promotion activity for 18,799 researchers active in the SNI program from 1991 to 2011, the analysis uncovers the following: while female researchers are associated with *more productivity* than males at each stage of the program, they are *less likely* to attain higher levels of promotion as they progress through their career. Illustratively, our more conservative results indicate women are associated with 1.2 more publications than men the year prior to promotion to Level 2 from Level 1 in SNI. Yet, 13 percent of women are associated with Level 2 promotion compared to 22 percent of men. To contextualize our understanding of these patterns, we interviewed SNI participants and include empirical assessments to unpack what may explain these perplexing results. While significant female representation in the applicant pool is needed to improve the gender gap, only a marginal increase in the gender representation of the reviewer pool is needed to reduce it significantly. This result points to a novel underexplored mechanism to inform future studies and policy – that of *evaluative salience*. While this does not fully address gender bias in the sciences, a shift in salience from applicants to reviewers may be an important precursor to address more structural ills around gender inequality.

## Introduction

Gender inequality in the sciences remains a persistent issue. Most notably, women are less likely to publish and receive less citations [1,2], less likely to patent [3,4], less likely to be principal investigators on grants [5,6] and less likely to engage in entrepreneurial endeavors that could result from their science [7,8]. This is not just detrimental to female scientists but also to scientific and social progress more generally. For instance, gender diverse teams can produce more impactful science [9], and the lack of patenting by women has negatively affected the rate of innovation in areas of significant societal consequence such as women’s health and technological development [10,11].

While the gap is closing on some of these metrics, such as publications and patents [12], there is still a well-documented “scissors effect,” whereby the gap is only closing among more junior ranks of academia (i.e., PhD students, post-doctoral scholars, and assistant professors) rather than in the more senior ranks (i.e., associate and full professors) [13]. Many studies reveal that while the number of women entering STEM fields has increased [1–8], gender discrepancies in publication rates persist, highlighting that men tend to publish more extensively than women both in terms of quantity and in high-impact journals. We observe similar trends in settings beyond the sciences, where women are more likely to work at the lower rather than upper echelons of the organization [14]. In short, the picture is a persistently stark one. Women are not able to participate in the scientific process as easily as men. And even when they do, this is largely constricted to opportunities at the lower rather than higher ranks of academia. These gender disparities have not just been a setback to female scientists but also to scientific and social progress, more generally.

Broadly, the trend of scholarship around R&D and scientific progress (including studies focused on gender discrepancies) is two-fold. First, most studies focus on scientific production in the Global North [15,16]. Second, studies most often focus on specific academic fields, especially the life and health sciences [11,13,17], or specific funding programs at distinct career stages [6,18]. Our study is groundbreaking for several reasons; namely, we: (1) extend the setting beyond the Global North; (2) execute an analysis that encompasses all STEM fields; (3) sample researchers from across an entire nation; (4) report comprehensive and detailed information on the publications and citations of each researcher; and (4) account for activity spanning 21 years of research output to track longer career trajectories (i.e., from junior to senior academic ranks).

To elaborate, we unpack these dynamics in a prominent national program in Mexico – Sistema Nacional de Investigadores (SNI). Not only does the structure of the program enable us to account for heterogeneity across career trajectories and across disciplinary boundaries, but also, prior scholarship on SNI documents an intriguing and counter-intuitive trend around productivity by gender. Gonzalez-Brambila and Veloso [19] used data on SNI researchers from 1991 to 2002 and found that gender gaps in scientific productivity were slight at an aggregate level (with women producing less than men). However, when focusing on citation trends, they report contrasting trends with women producing greater impact in the areas of engineering, social sciences, and humanities. Rivera-Leon et al. [20] report similar trends. Tracing SNI researchers in 2013 from the broad fields of mathematics, physics, chemistry, and medicine, Rivera-Leon and colleagues found that the average female researcher in public universities is around eight percent more productive than her male peers. Together, this scholarship seemingly contradicts the prevailing narrative of men outproducing women. We examine this counterintuitive puzzle in extended ways that more fully leverage the program’s heterogeneity, rather than just aforementioned aggregated and averaged effects by gender that do not unpack disciplinary and career stage variation.

Drawing from comprehensive descriptive trends of the national program and illustrative qualitative data, we find that female researchers are associated with *more productivity* than males. Beyond replicating this finding, we also show that women are *less likely* to attain higher levels of promotional certification in SNI as they progress through their career. While recent scholarship points to some closure in the productivity gap [21–24], this trend in the Mexican context is distinct and surprising. Prior scholarship attributes lower productivity for women due to limited access and opportunities and more administrative burdens [25,26]. However, in our setting, women surpass men in terms of productivity and yet they still do not see the same promotional opportunities. The remainder of this paper seeks to explore and unpack this perplexing finding.

## Empirical context

Our study focuses on Mexico’s National System of Researchers or the Sistema Nacional de Investigadores (SNI) program. An OECD review and assessment of Mexican innovation policy emphasized SNI’s success in stimulating productivity of researchers in Mexico, especially in increasing the number of scientific publications and citations [27]. Fundamentally, this program was designed to enhance the international recognition of Mexican researchers and bolster the quality of the research base and the attractiveness of research careers in the country.

### Program overview

Formally, SNI was created in 1984 and was established in part to respond to the 1982 economic crisis when many researchers left Mexico for economic opportunities abroad. SNI first worked to establish compensation packages to ensure greater retention, but since then, the program has also served as a mechanism to enhance research productivity [28,29].

Program beneficiaries are researchers who are systematically engaged in research activities and have either a research contract or an institutional agreement with a higher education institution or research center in Mexico’s public, private, or social sectors. Foreign researchers can also become SNI members provided they work at a Mexican higher education institution or research center for at least one year before applying to the program.

The National Council for Science and Technology (CONACYT) oversees the administration of SNI. Researchers can apply for membership (i.e., entry, re-entry, continuation, or upgrading of level) through an annual open call issued by CONACYT. In addition to financial compensation, being a member of SNI provides professional prestige and is usually a prerequisite for academic promotion and access to public research grants.

Formally, SNI historically covered seven scientific areas (as of 2022, there are nine areas of knowledge) and four promotional levels of certification. The program oversees activity in the following academic areas: Physics, Mathematics & Earth Sciences (Area 1); Biology & Chemistry (Area 2); Medicine & Health Sciences (Area 3); Humanities & Behavioral Sciences (Area 4); Social Sciences (Area 5); Biotechnology, Agriculture & Livestock Sciences (Area 6); and Engineering (Area 7). Moreover, SNI designates four academic ranks or “Levels of distinction.” This includes Candidate (L0 – beginner), Level 1 (L1 – junior), Level 2 (L2 – intermediate), and Level 3 and Emeritus (L3 – senior) scientists. Admission into each level is accompanied by a commensurate and tax-free monthly stipend that may represent up to 30 percent of the researcher’s total income [19]. The higher the level of promotional rank, the higher the pecuniary compensation.

Researchers are predominantly evaluated on their productivity, though other evaluation metrics become important as one seeks higher promotion. Productivity is measured in the form of published articles, books, research results and book chapters, as well as patents, technological innovations, and technology transfer, if applicable. For the academic fields of Physics, Mathematics & Earth Sciences (Area 1), Biology & Chemistry (Area 2), Medicine & Health Sciences (Area 3), Biotechnology, Agriculture & Livestock Sciences (Area 6), and Engineering (Area 7), publications in Web of Science are considered the most important metric of productivity. Additionally, evaluations also include teaching and mentoring activity in terms of the number of professional and post-graduate advisees, the number and evaluation of undergraduate and graduate courses, and the formation of new researchers and research groups. Other service criteria include participation in scientific and technology councils, editorial bodies, and scientific committees and participation as a technical evaluator in grants supported by CONACYT. This latter measure reflects one’s national and global scientific reputation.

Table 1 details the expectations and deliverables for each level in addition to the corresponding compensation package. Most notably, as researchers rise in rank across the levels, they must demonstrate increasing quantity and quality of research, teaching, and service impact. Moreover, SNI has been engaging in reforms to address the diversity of composition in Mexico’s research base over time. In 2000, SNI reformed its regulations for pregnant researchers. Upon request, women are eligible for a one-year extension.

**Table 1:** SNI certification levels and compensation.

	*Research expectations*	*Teaching expectations*	*Service Expectations*	*Compensation Package*
*Candidate (beginner)*	• Doctoral degree completion or ability to perform original research in their field. • At least one published article in an indexed journal or chapter as first author	• N/A	• N/A	3 minimum salaries (432 USD per month)
*Level 1 (junior)*	• Established and defined line of research • Published book or five articles, book chapters or research maps	• Demonstrate participation in teaching tasks and in training of human resources	• N/A	6 minimum salaries (864 USD per month)
*Level 2 (intermediate)*	• Meet expectations of Level 1 • Demonstrate consolidated research career (including publishing of books, articles, book chapters, reviewer, critiques, coordinated books, anthologies or compilations and editions or relevant translations	• Meet expectations for Level 1 • Successful mentoring of undergraduate and/or highly qualified human resources	• Achieved a new research line or agenda	8 minimum salaries (1,152 USD per month)
*Level 3 (senior)*	• Meet expectations of Level 2 • Established recognized contribution to knowledge • Published works of significance in their line(s) of research	• Meet expectations of Level 2	• Attain international leadership and prestige, demonstrated through academic recognition or distinctions, quality citations to their works, and media reviews	14 minimum salaries (2,016 USD per month)

Researchers in institutions in all states (except Mexico City) receive an additional minimum salary. Those in Mexico City receive one less, respectively. A minimum salary is the lowest remuneration for employees, and compromises 144 USD/month equivalent (exchange rate in 2012).

To manage the certification status and promotion, SNI appoints seven review committees – one for each of the seven general areas of knowledge. Each review committee comprises 14 eminent scholars who have attained Level 3 certification in that respective area. Starting in 1997, if there are no Level 3 researchers in a specific discipline, a peer committee could identify one from the set of Level 2 researchers. The review committees devise and revise specific criteria for evaluating the performance of their colleagues in each respective area according to their area’s distinct methods and traditions. Based on a merit-based scheme, the SNI review committees assess applications annually and decide which certification level the applicant should enter, as well as whether to renew or upgrade an applicant’s current certification level. If a researcher disagrees with the committee’s verdict, the researcher can formally appeal to a separate appeals committee. All seven review committees are partially changed on an annual basis with the objective of maintaining a mix of new and prior committee members in the same group.

## Quantitative analysis

### Methods for quantitative analysis

To understand trends of the national program, we trace programmatic activity for the entire population of SNI recipients across the breadth of STEM fields from 1991 to 2011. Of note, the former date marks the initial year of comprehensive data availability for the program. From January 15–20, 2012, we pulled administrative detail from the CONACYT primary web page and archived sources [30,31]. The population consists of all researchers in SNI for at least one year during this timeframe. Then, we added data from the Web of Science (WoS) to trace the number of publications and citations for each researcher in the program during this time. Given the archival nature of the publication data, we obtained data from 1991 to 2002 from a national citation report [32]. For publication data from 2003 to 2011, we accessed the WoS public online platform. Altogether, we pulled WoS data between June 25 to December 10, 2012. Thereafter, we precisely matched the two sources of SNI activity and WoS publication activity using the scholar’s name.

Importantly, we extend the study timeframe 21 years; this enables us to trace both aggregate trends across all four promotional levels (Candidate (L0), Level 1, Level 2, and Level 3), in addition to individual researcher progression through SNI over the course of their own professional careers. With a focus on STEM, we narrow the sample to the following five areas – Physics, Mathematics, & Earth Sciences (Area 1); Biology & Chemistry (Area 2); Medicine & Health Sciences (Area 3); Biotechnology, Agriculture & Livestock Sciences (Area 6); and Engineering (Area 7). We exclude the social sciences and humanities (Areas 4 and 5) as these academic areas follow different research productivity norms that include book manuscripts, which markedly differ from the areas that are more reliant on WoS-based publication criteria. In sum, this comprises 18,799 total researchers; 72 percent are male and 28 percent female.

### Descriptive statistics

Table 2 reports descriptive statistics for the sample of SNI recipients and includes comparison of means contrasting trends by gender. The majority of SNI recipients received their PhD from a Mexican university (62 percent); however, men are more likely to receive their training from US and European institutions. The two areas of Biology & Chemistry (Area 2) and Engineering (Area 7) account for the largest share, though women disproportionately lie in the former area and men in the latter. Moreover, across the other STEM fields, we observe discrepancies in gender distribution with women accounting for a disproportionately greater share in Medicine & Health Sciences (Area 3; 22 percent of women or 1,161 women) and men accounting for a disproportionately greater share in Physics, Mathematics, & Earth Sciences (Area 1; 23 percent of men or 3,110 men).

**Table 2:** Descriptive statistics.

	(1) Mean	(2) S.D.	(3) Female	(4) Male	(5) t-stat	(6)
Male	0.72	0.45	0.00	1.00	.	.
PhD Age	34.74	5.89	34.86	34.69	1.69	*
Mexican PhD	0.62	0.49	0.73	0.57	19.48	***
European PhD	0.22	0.42	0.18	0.24	−8.67	***
USA PhD	0.12	0.33	0.07	0.14	−15.53	***
Other PhD	0.04	0.19	0.02	0.04	−6.60	***
Physics, Math & Earth Sciences	0.21	0.40	0.14	0.23	−16.02	***
Biology & Chemistry	0.23	0.42	0.34	0.19	20.51	***
Medicine & Health Sciences	0.14	0.34	0.22	0.11	17.32	***
Biotech & Ag Sciences	0.19	0.39	0.17	0.20	−4.82	***
Engineering	0.23	0.42	0.14	0.27	−21.76	***
States with more economic development	0.84	0.37	0.87	0.83	5.31	***
Large Institution	0.31	0.46	0.35	0.29	7.65	***
Age at reaching Candidate (L0)	34.35	4.33	34.38	34.34	0.40	
Age at reaching L1	40.35	7.27	40.19	40.41	−1.57	
Age at reaching L2	47.18	7.68	48.64	46.84	5.49	***
Age at reaching L3	52.97	8.33	54.99	52.66	3.56	***
As far as L0	0.31	0.46	0.34	0.30	5.03	***
As far as L1	0.49	0.50	0.53	0.47	7.29	***
As far as L2	0.13	0.34	0.10	0.15	−9.93	***
As far as L3	0.06	0.25	0.03	0.08	−14.16	***
Unique Researchers	18799		5279	13520		

Col. 1 and 2 report descriptive statistics for full sample (all researchers in SNI reporting at least one year of activity between 1991–2011). Col. 3–6 report comparison of means between men and women. ***p < 0.01, **p < 0.05, *p < 0.1.

**Fig 1:**
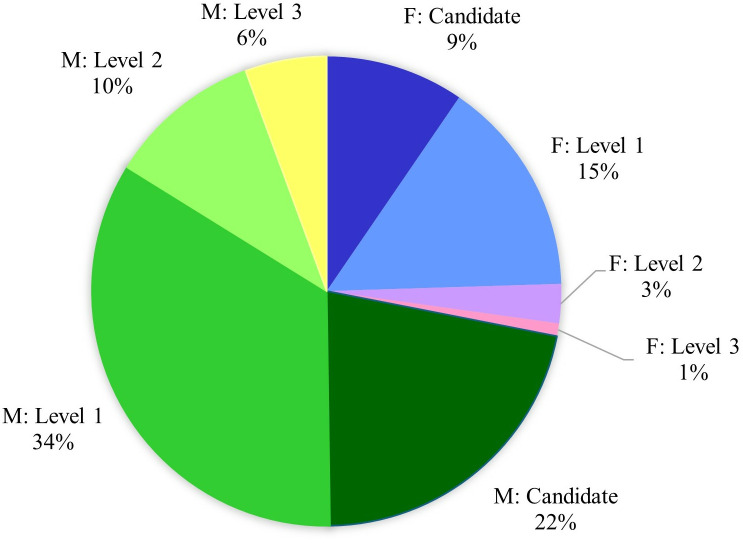
Distribution of SNI participants by rank & gender. Image depicts distribution of SNI population over timeframe of study, 1991–2011, by promotional rank and gender. M denotes males, F denotes females. We report proportional for all four ranks (Candidate, Level 1, Level 2, and Level 3).

**Fig 2:**
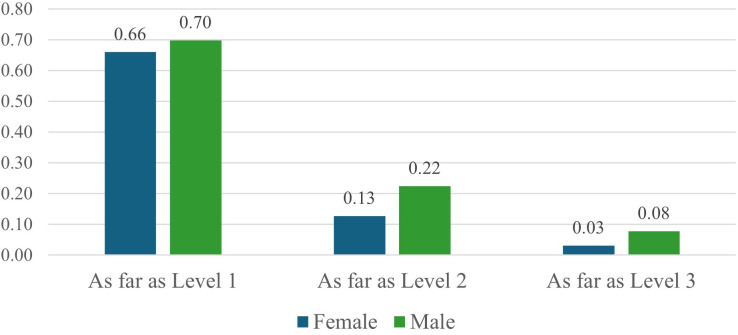
Promotion rates (by rank and gender). Image reports promotion rate by rank and gender. Estimates report aggregate trends over timeframe of study, 1990–2011.

To assess promotional paths across the various levels of the SNI program, we report additional detail in Fig 1 and Fig 2. Together with Table 2, we observe the following. On average, men and women are comparable in achieving Candidate (L0) rank by age 34 and Level 1 rank by age 40. However, for the later-stage promotional milestones, men achieve higher ranks at a faster pace. At this stage, they secure Level 2 roughly two years earlier than women at age 47 and they secure Level 3 roughly one and half years earlier than women at age 53. Furthermore, Fig 1 illustrates the overall distribution of research rank highlighting disparities in achievement by gender. While men account for a greater share of the SNI researcher population, they also account for a disproportionately greater share of achievement at higher promotional ranks (i.e., Level 2 and Level 3) compared to women. On average, 13 percent of women ever achieve the higher ranks of Level 2 and/or Level 3, while 23 percent of men ever achieve such status (refer to Table 2). S1 and S2 Figs in S1 File report dynamic activity for these trends as well. Gender discrepancies persist, however, the trends by rank are balancing over the more recent years in the timeframe.

Fig 2 reports further detail on promotion rates across the program. Men and women report proportionately comparable transition rates in achieving rank of Level 1–66 (70) percent of women (men) achieve such status. However, we observe more pronounced discrepancies at the higher ranks of Levels 2 and 3 with women transitioning at proportionately lower rates. Specifically, 13 percent of women achieve Level 2 status compared to 22 percent of men, while 3 percent of women achieve Level 3 status compared to 8 percent of men. Altogether, these descriptive statistics indicate that men not only account for a greater share of the SNI population, but they also are promoted through the program to higher ranks (i.e., from Candidate to Levels 1, 2, and then 3) at faster and greater rates than women.

Referencing back to the “scissors” analogy introduced at the beginning of this study, the blades are closing but the scissors remain open; while the gender gap is closing at the lower academic ranks, the gap remains at the higher ranks. Based on the SNI criteria for promotion in rank, this would suggest that men outperform women in terms of research productivity. We now examine this feature of the program.

### Productivity trends

**Fig 3:**
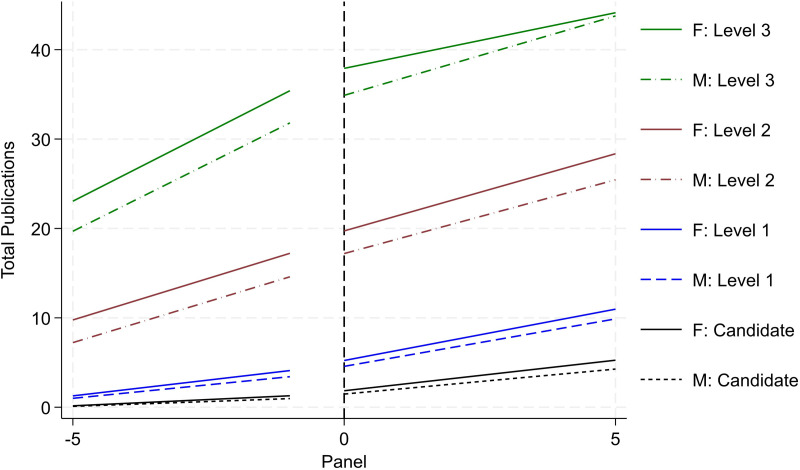
Publication productivity by rank and gender (status – ever achieve). Image reports aggregate productivity trends of cumulative publication count. We report four sets of two trends. Solid lines denote female activity. Dotted lines denote male activity. Each set is designated by a separate color scheme and reflects the four promotional ranks, respectively. Black denotes Candidate; blue denotes Level 1; red denotes Level 2, and green denotes Level 3. For each set, we trace productivity over the five years leading up to the promotion and the five years following. To identify the sub-sample for each set, we draw from the entire population of SNI and focus on activity around the respective promotion.

**Fig 4:**
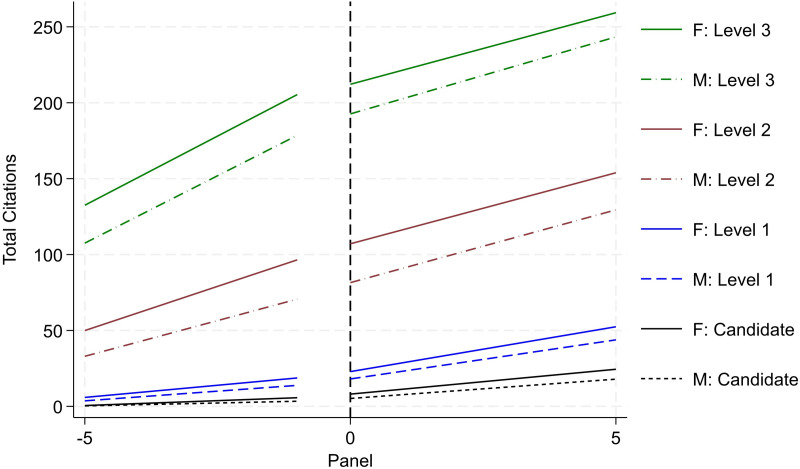
Citation productivity by rank and gender (status – ever achieve). Image reports aggregate productivity trends of cumulative citation count. Refer to notes in Fig 3 for all other detail.

Figs 3 and 4 report researcher productivity trends by gender and rank (i.e., Candidate, Level 1, Level 2, and Level 3). Operationally, we re-center the timeframe for each researcher based on when they initially secured the various ranks in the SNI program. This enables us to trace the quantity and quality of research productivity over a standardized timespan (i.e., the five years leading up to promotion followed by the subsequent five years). For research quantity, we examine cumulative peer-reviewed publications (reported in Fig 3); and for research quality, we examine cumulative citations (reported in Fig 4). All publishing data is drawn from the WoS. For each rank, we draw from the population of researchers that ever achieved the various milestones. The solid lines report productivity for women and the dotted lines report for men. Black trends denote activity for Candidates; blue denotes trends for Level 1; red denotes trends for Level 2; and green denotes trends for Level 3.

A consistent and clear trend emerges. When evaluating the trends within rank by gender, on average, women produce higher levels of research output and impact than men. This trend holds both before and after the SNI promotion and across the various ranks. This aligns with prior scholarship on the SNI program reporting greater productivity for women than men [20]. Moreover, the size of the difference in productivity increases across the more senior ranks. For example, looking exclusively at pre-trend differences (i.e., at the time when the researcher is being evaluated by the review committee), women are associated with one-third more publications than men the year prior to being promoted to Candidate. Across higher ranks, this difference increases to a 0.69 publication differential for Level 1, a 2.51 publication differential for Level 2, and a 3.61 publication differential for Level 3. These differences persist in the various post periods as well.

While the results from Fig 3 and Fig 4 highlight that women outperform men in terms of research productivity, recall that Fig 2 – reporting promotional transition rates by rank and gender – reports *contrasting trends* with men being promoted at higher rates than women. Together, this set of results presents a puzzle. Formally, promotion in the SNI program is merit-based with greatest emphasis placed on research productivity (namely, publications). *Yet, we observe that women are promoted at* ***lower*** *rates than men despite* ***outperforming*** *men in terms of research quantity and quality*. To understand this discrepancy, we unpack these results further. Namely, we assess the extent to which selection, history, heterogeneity, and institutional support may drive or impact the overall trends around this productivity-promotion puzzle. We discuss each in turn.

#### Selection

**Fig 5:**
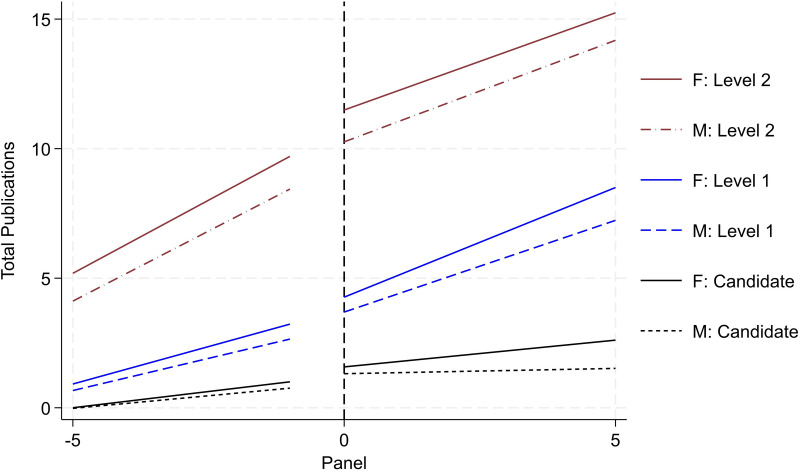
Publication productivity by rank and gender (status – as far as). Image reports aggregate productivity trends of cumulative publication count. To identify the sub-sample for each set in this image, we redefine various samples by their highest rank achieved (i.e., as far as Candidate; as far as Level 1; as far as Level 2). Refer to notes in Fig 3 for all other detail.

**Fig 6:**
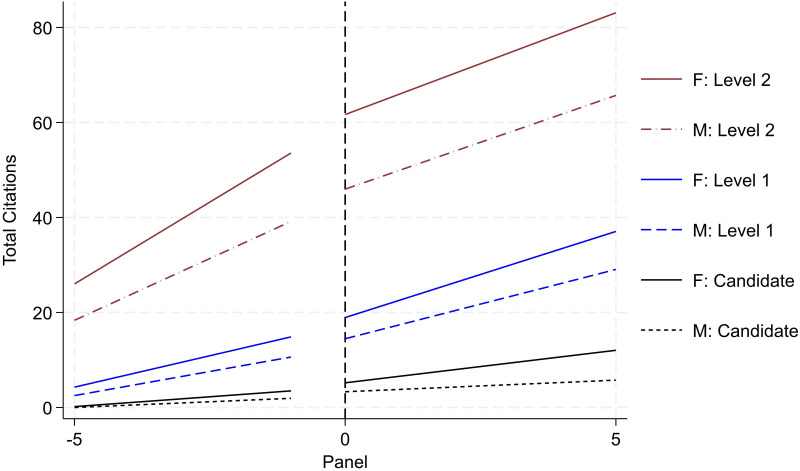
Citation productivity by rank and gender (status – as far as). Image reports aggregate productivity trends of cumulative citation count. Refer to notes in Fig 5 for all other detail.

Not all researchers are promoted across the four ranks. Attrition persists across the sample with some researchers only achieving Candidate, Level 1, or Level 2 rank; moreover, leading scholars (with disproportionately high levels of research output) may skew the results (albeit in the opposite direction from the attrition set). Altogether, the variation in each researcher’s professional trajectory may skew the results. To improve comparison in latent ability and academic achievement, we redefine various samples by their highest rank achieved (i.e., as far as Candidate; as far as Level 1; as far as Level 2). We report such research productivity trends by gender and rank in Fig 5 and Fig 6. We do not include Level 3 since the “ever Level 3” reported in Fig 3 and Fig 4 and “as far Level 3” are the same. We observe similar (albeit attenuated) trends as reported in Fig 3 and Fig 4, respectively. Women are associated with 0.25 more publications than men the year prior to being promoted to Candidate; this difference increases to a 0.58 publication differential for Level 1, and a 1.20 publication differential for Level 2. We argue that this sample of the “as far as” designation provides a more valid sample for comparison than the “ever” designation, and we use this specification henceforth for analysis.

Before consideration of other potential confounding factors (i.e., history, heterogeneity, and institutional support), S3 Fig in S1 File teases apart selection one step further. Recognizing that researchers progress to various ranks and attrition in the program persists in various forms, we provide a series of four-way comparisons by each promotional rank. Using the Candidate rank as an illustrative exemplar, we include men and women that only progress as far as Candidate. These two groups account for two of the four comparisons and are denoted by dotted black and blue lines, respectively. In addition, in the same panel we include men and women that progress only as far as the *next highest* rank (i.e., Level 1), but we trace their productivity levels around their promotion to Candidate. These two groups account for the latter set of comparisons and are denoted by solid back and blue lines, respectively.

In the case of Candidates (Panel A of S3 Fig in S1 File), those that ultimately progress to the next higher rank outperform those that progress to the referent rank, though the differential trend by gender persists – women outperform men in both comparisons. On average, women that ultimately achieve Level 1 are associated with approximately 0.40 more publications than men with similar trajectories the year prior to being promoted to Candidate; women that only achieve Candidate rank are associated with 0.25 more publications than their male counterparts the year prior to being promoted to Candidate. The results in both Panel B and Panel C of S3 Fig in S1 File (tracing activity around Level 1 and Level 2 promotion) illustrate a different trend with more persistent gender discrepancies reporting women with higher output. Focusing on the promotion of Level 1, while women outperform men on average, women that only progress to Level 1 produce *more than both sets of men*, including those that are promoted to Level 1 and ultimately to Level 2 (this holds across both the pre and post trends).

In short, even when accounting for various paths of attrition through the program, women outperform men on average, and yet they are nonetheless promoted at lower rates. Moreover, prior to the point of promotion, women who do not proceed to the next level often are nearly equivalent to men who are promoted (i.e., Candidates) and even arguably surpass men when assessing the higher promotional ranks (Level 1 and Level 2).

#### History

Given the 21-year timeframe, we assess the extent to which history may confound the effects as the composition of SNI participants shifted. Most notably, the participation of women increased overtime (as reported in S1 and S2 Figs in S1 File). S4 Fig in S1 File provides insight into potential dynamics. Following the same format as Figs 5 and 6, Panel A of S4 Fig in S1 File reports productivity trends among researchers that entered the SNI program before 2001 (the year that designates a significant administrative shift for the program, including revisions for greater maternity support [33]) while Panel B of S4 Fig in S1 File reports productivity for entry thereafter. Where we observe differences (especially in the pre-period leading up to promotion), women outperform men, though the differences attenuate in the more recent panel. The more recent activity in the program points to a greater closure in the gender gap. As we explore in the Mechanisms section below, we consider whether this may be due to increasing representation of women in the reviewer and applicant pool over time.

### Academic heterogeneity

Accounting for the breadth in research norms by discipline [34], we distinguish the various academic areas that comprise the STEM fields. S5 Fig in S1 File reports this detail. Of note, researchers at various ranks in Medicine & Health Sciences (Area 3), Biotechnology, Agriculture & Livestock Sciences (Area 6), and Engineering (Area 7) drive the overall effect of women outperforming to men. For the other areas, we report trends complementary to prior scholarship [19] and observe greater comparability in research productivity and even trends where men outperform women (i.e., Physics, Mathematics & Earth Sciences (Area 1) and Biology & Chemistry (Area 2)).

### Institutional variation

With a focus on training and placement, we distinguish between researchers that received their PhD domestically from a Mexican institution verses a foreign institution (S6 Fig in S1 File), and, separately, between those working at large versus smaller institutions (S7 Fig in S1 File). Both sets of moderators may impact a researchers’ publication trajectory. However, with minimal exception, we observe similar results as reported in Figs 3,4,5, and 6 – women outperform men over time across rank, including at the time when the researcher is under consideration for promotion.

Altogether, these results report a consistent trend – on average, women outperform men in terms of research productivity, yet they are promoted in the SNI program at disproportionately lower rates. We additionally note that this gap declines over time. On the one hand, this suggests SNI promotion may be driven by a set of review criteria that is not captured by research output. On the other hand, the discrepancies in these trends may point towards gender biases, placing women at a disadvantage. To contextualize our understanding of these patterns, we conducted a series of interviews with SNI participants and include additional empirical assessments to unpack the mechanisms that may explain these puzzling findings.

## Qualitative analysis

### Methods for qualitative analysis

To gain more granular insight into this program, a member of our co-authoring team conducted eight virtual interviews between May 22 and June 10, 2024 with researchers that are currently or were once in the SNI system. To have a diverse representation of researchers, we sought to gather an even distribution of male and female participants from a wide swath of SNI scientific areas and institutional affiliations (i.e., public and private higher education institutions and research centers of varying size). Moreover, we sampled researchers from different states, including and beyond Mexico City. Each interview lasted 15–60 minutes. When conducting the interviews, we provided each interviewee with a privacy notice (as formally drafted by the institution’s General Attorney). We received verbal acceptance of the privacy notice at the beginning of each interview. Recorded interviews were conducted in Spanish and then translated to English. The interviews were open-ended, and we asked recipients about their impressions on the evaluation process of SNI, their research outputs, and their reflections of the changes on productivity with rank changes. To conclude, we asked the interviewee about gender differences in the evaluation process.

### Qualitative results

According to our interviewees, SNI aims to promote a holistic approach to research that includes student training, teaching, outreach, and the production of high-quality written work (i.e., publications), emphasizing consistency and international recognition. For instance, although journal impact factors are not officially used as criteria for evaluating research performance, some academic areas (e.g., Biology & Chemistry (Area 2)) have internal criteria requiring articles to be published in journals with specific impact factors. Evaluations also focused on the quality, consistency, and coherence of research activities, leadership, and international recognition. The quality of research outputs is assessed based on their originality, influence on human resource training, and impact on solving scientific and technological problems. This all suggests a departure from purely quantitative productivity metrics (i.e., citations and publications) to more subjective criteria that reflect notoriety, especially as one goes up certification levels. As one interviewee noted:

Level 1 researchers are measured by valid products. You can remain at Level 1 your whole life if you consistently produce valid products. At Level 2 what matters is in which journals researchers publish, how many citations they receive, and what impact factor the products have. Level 3 is about the long-term impact of that work, measured more qualitatively (h-index, citations, conferences, editorial roles, invitations to conferences).

In fact, some even felt such increased subjectivity was a necessity. As another respondent noted, “*It’s important to differentiate that Level 1 is measured by quantitative criteria such as the number of products, while at Level 3, the numbers no longer matters but rather the impact (qualitative).*”

Moreover, evaluation criteria are tailored to the relevant academic products for each discipline, with individual performance compared to the average performance within that discipline, rather than scientists competing directly with one another. This requires nuance not only across different areas but within areas (S8 Table in S1 File reports productivity by discipline within academic area to illustrate this claim). As one interviewee noted about Physics, Mathematics, & Earth Sciences (Area 1), “*Evaluations in Area 1 are different depending on whether you’re a mathematician, physicist, astronomer, etc.*” Nonetheless, a researcher is usually rejected from SNI if they do not have at least three publications over three years, which remains a key element for determining SNI level.

Given this need for tailoring across and within areas to ensure the most appropriate comparison (though some general metrics seem to apply), as well as the introduction of more subjective criteria as one goes up certification levels, interviewees noted this led to a highly complex evaluation process. For instance, how to combine and weight qualitative and quantitative factors was especially unclear. As another researcher commented, “*I agree evaluations should be more qualitative, but it’s difficult not to take productivity into account in a more quantitative way*.” While some argued there were clear metrics that could indicate scientific advancement (i.e., total funded projects, total students, etc.), the combination of quantitative and qualitative factors led to ambiguity as to what level to confer on applicants. One researcher emphasized, “*Moving up levels is a very complicated issue because evaluators face the challenge of deciding which level to place the researcher.*”

Surprisingly and despite the fact prior studies note that ambiguous and subjective criteria often harm female researchers [35], no one interviewed perceived gender differences in the evaluation criteria at SNI. Rather, reactions around gender could be characterized in two ways. The first was seeing gender biases as more socially systemic across institutions. As noted, *“I haven’t seen that women need to do more things; however, I do believe that everything is more challenging for them due to how gender roles are in society, not in the institutions themselves*.*”* The second, though, was an acknowledgement of important differences in the proportion of women in some areas of knowledge. For instance, the proportion of women in Physics, Mathematics & Earth Sciences (Area 1) is relatively small. This directly impacts the potential representation of women on the corresponding review committee. As noted, *“There weren’t as many women as men. In physics, our rule was that the review committee should have the same proportion as the community; that is, if a community is 20 percent women, the committee must also have 20 percent women.”* Given the scarcity of women in the highest level of SNI, the review committees have been predominantly male. This could impact the review process.

Overall, the interviewees recognize the complexity of programmatic oversight and administration. SNI review requires not only quantitative, but also qualitative aspects especially at higher certification levels. Yet interestingly, there was a general sentiment among the interviewees that gender inequities in academia were no different than other parts of society, despite acknowledging fewer women were present in the SNI program.

## Mechanisms

We couple insights across our qualitative interviews and descriptive results with the prior literature to assess mechanisms that may explain why we are generally observing both higher productivity and yet less advancement among female researchers. First, we consider the gender composition of the review committee. Prior scholarship documents the persistent role of homophily, especially in terms of peer review, whereby individuals are more likely to favorably review peers with similar backgrounds than those with differences [36–39]. Hence, we anticipate that increasing the representation on female researchers on the review committee may diminish the gender gap in promotion.

**Fig 7:**
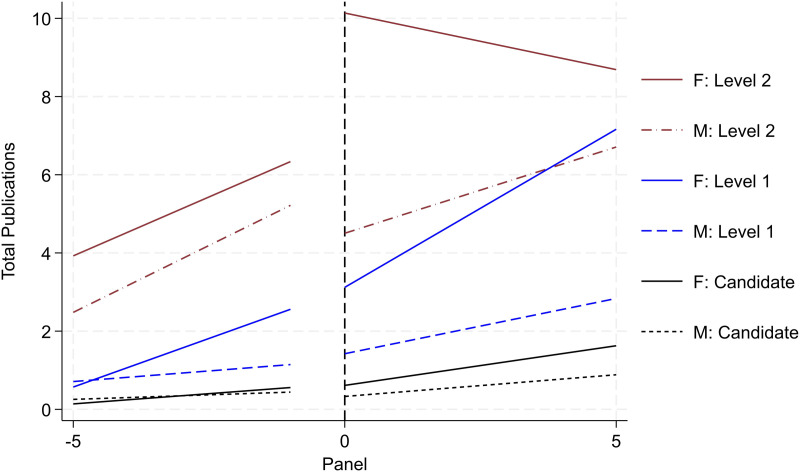
Publication productivity based on gender diversity of reviewer pool – likelihood of women on review panel (0 percent). Image illustrates heterogeneity of trends reported in Fig 5 based on the likelihood of the gender composition of the review committee – likelihood of women on review panel (0 percent). We infer the gender composition based on the share of female researchers in Level 3. We derive this composition by area and year and focus on the year each researcher is under consideration for a given promotion. Refer to notes in Fig 5 for more detail on formatting.

**Fig 8:**
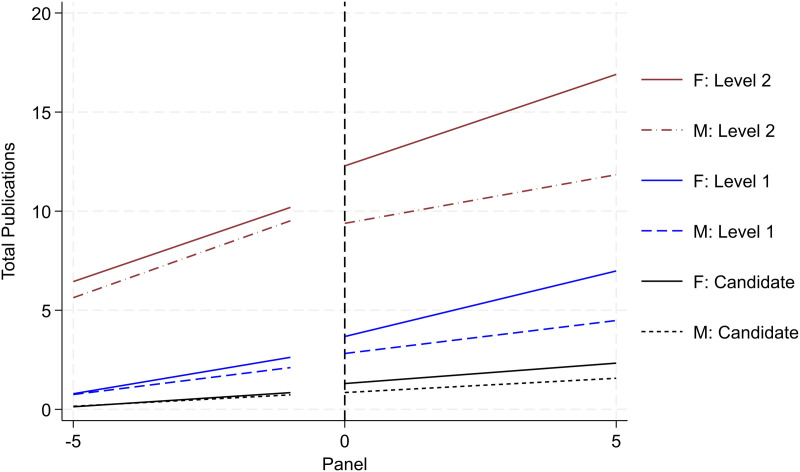
Publication productivity based on gender diversity of reviewer pool – likelihood of women on review panel (5 percent). Image illustrates heterogeneity of trends reported in Fig 5 based on the likelihood of the gender composition of the review committee – likelihood of women on review panel (5 percent). Refer to notes in Fig 7 for more detail.

**Fig 9:**
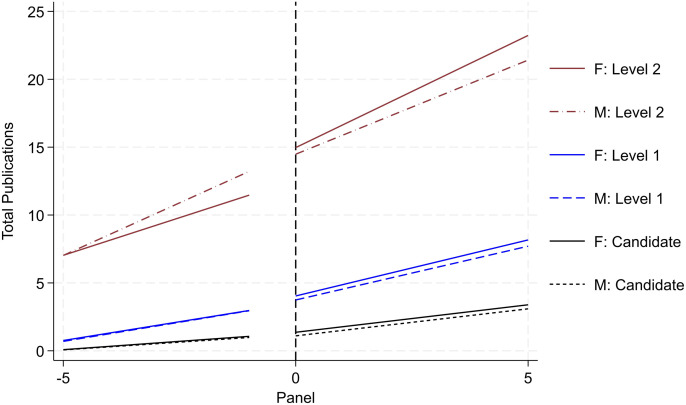
Publication productivity based on gender diversity of reviewer pool – likelihood of women on review panel (10 percent). Image illustrates heterogeneity of trends reported in Fig 5 based on the likelihood of the gender composition of the review committee – likelihood of women on review panel (10 percent). Refer to notes in Fig 7 for more detail.

To assess this trend, we make a reasonable assumption from administrative records and our qualitative interviews that the aim is to have review panels reflective of the scientific area’s gender composition. Recall, every year SNI selects the review committee by area based on the pool of Level 3 researchers in that area [40]. While the detail on the history of the review panels is not publicly available, we can approximate a potential gender composition of the committee based on the ratio of women with such rank (by area and year). Figs 7, 8, and 9 report the research productivity trends by gender and rank across various designations based on the likelihood of women serving on the review panel. Fig 7 includes the population of researchers where the likelihood of women on the review panel is zero (i.e., there were no women (by area) with Level 3 rank in the year the researcher went up for promotion). Figs 8 and Fig 9 report increasing likelihoods of women on the review panel by 5 and 10 percent.

A distinct trend emerges. The differential effect in productivity between women and men is most pronounced (with women outperforming men) where no women could have served on the review panel. The effect attenuates as the likelihood of women on the review panel increases (Fig 8) and effectively diminishes once the likelihood of women on the review panel reaches 10 percent (Fig 9). In fact with 10 percent increased likelihood of women on the review panel, men outperform women in the year leading up to Level 2 promotion. In other words, the differential research expectations for promotion for women attenuate as the likelihood of women serving on the review panel increases.

Second, we explore how gender composition of the applicant pool may impact promotion rates. Again, based on the qualitative insights and detail from the quantitative trends reported here and in prior studies [19,20], the share of women by area is disproportionately unbalanced. Greater diversity in the applicant pool likely normalizes and balances research expectations for women [39,41,42]. Conversely, women may face greater discrimination if they are among a smaller minority in the applicant pool.

**Fig 10:**
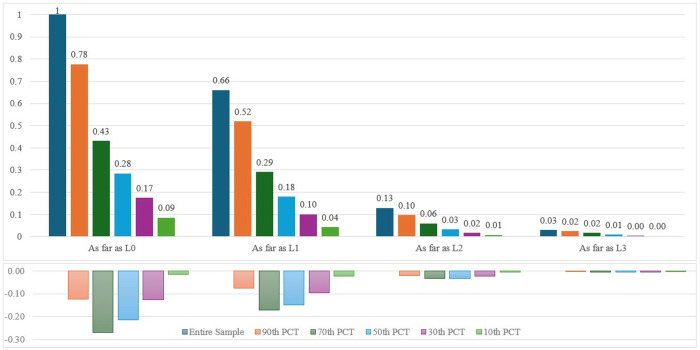
Promotion rate for women based on gender composition of applicant pool. We split the sample into four sub-groups based on the ultimate rank achieved (i.e., as far as Candidate, as far as Level 1, etc.). In the top portion of the image, we report the promotion rate based on further sub-samples as designated by the gender composition of the applicant pool. The dark blue bar depicts the transition rate for the entire sample of women (mirroring the detail reported in Fig 2); the green bar reports the transition rate for women whose applicant pool of women only includes up to the tenth percentile distribution. We subsequently increase the applicant pool of women up to the 30^th^ (purple bar), 50^th^ (light blue bar), 70^th^ (dark green bar), and 90^th^ (orange bar) percentile distributions. The bottom portion of the graph reports the differential between the actual promotion rate and the assumption that the transition rate by percentile distribution scales at a linear rate. The negative differential reflects the level of gender bias in the promotion process.

Fig 10 reports the transition rate for women across various sub-samples. Namely, we designate samples based on varying compositions of the applicant pool. Operationally, we compute the percentile distribution of the gender ratio of the applicant pool by area and rank for the year each woman is up for promotion. The blue bar depicts the transition rate for the entire sample of women (mirroring the detail reported in Fig 2); the green bar reports the transition rate for women whose applicant pool of women only includes up to the tenth percentile distribution. We increase sample subsequently by increasing the applicant pool of women up to the 30^th^ (purple bar), 50^th^ (light blue bar), 70^th^ (dark green bar), and 90^th^ (orange bar) percentile distributions.

Here, again, a clear trend emerges. The transition rate by promotional level decreases as the composition of women in the applicant pool decreases. We observe this across each rank. Though, to place this in greater perspective, we interpret this trend with the following assumption. If gender were not a factor, then the transition rate by percentile distribution would scale at a linear rate. For example, the transition rate to Candidate (L0) for women in the 90^th^ percentile distribution would be 0.9 (i.e., 90 percent of the entire sample); however, we report a transition rate of 0.78. We estimate the differential from the linear rate (assuming no gender bias) to the actual transition rate in the bottom half of the image. This negative differential illustrates that women are promoted at a disproportionately lower rate as the composition of the applicant pool decreases. In S2 Fig in S1 File and as anticipated, the historical closure of the gap does indeed seem largely attributed to these increasing proportions of women in both the reviewer and applicant pool.

## Discussion

This study offers two key insights that diverge from prior scholarship on gender, R&D, and productivity. First, in tracing programmatic activity in SNI, we report that women are associated with *more productivity* than men, and yet they are *less likely* to get promoted. Second, this disparity has *reduced* over time as female representation at the higher ranks has *marginally increased*. In other words, the blades of the scissors seem to be closing but not in the ways that we expected.

The prevailing literature largely reports countervailing findings to our results. Namely, a litany of studies document that women are less productive than men [1–8]. This has been detrimental to general social and scientific progress [9–11]. Moreover, it includes clinical implications – when productivity is disproportionate across gender, the direction of research shifts towards male-dominated issues and away from female-dominated ones [11,43]. Scholars argue that asymmetric administrative burdens [44,45], all the way to outright discrimination and bias [46–48], account for the productivity gap. If we consider recent insights in the literature around corporate board gender representation, the prevailing view is that minimal representation is inadequate to solve these issues. In fact, studies show that boards increasingly have two women to avoid perceptions of tokenism. Yet increased representation drops precipitously thereafter, suggesting representation is still about appearances rather than substance [49]. Taken together, the overall implication from this prior scholarship is that women experience asymmetric administrative burdens and structural barriers to opportunities that prevent them from being productive. And even having some women in the highest academic ranks is inadequate to address these inequities.

Our results uniquely contribute to this discourse by examining a broader range of scientific fields (i.e., beyond a single area of science), professional ranks (i.e., junior and senior faculty), and social context (i.e., Mexico). Contrary to previous scholarship, we report that women are associated with *greater* productivity, and yet they still face *lower* promotion rates. Moreover, while a large increase in the applicant pool is needed to attenuate the gap, only a *marginal increase* in the gender representation of the *reviewer* pool is needed to close it.

To reconcile our findings with existing literature, we triangulate our quantitative data with qualitative interview insights [50]. Interestingly, both women and men do not sense such gender disparities. Julia Tagüena, former leader of UNAM’s (National Autonomous University of Mexico) General Directorate of Science Popularization, publicly echoed this sentiment [51]. Few appeal rejection decisions from SNI (even when most appeal cases are successful). This reflects a notion that program recipients seemingly believe such decisions made by the review committee are fair and impartial. Combining these insights with the various marginal shifts in female representation (refer to Figs 7,8,9, and 10) suggests a novel underexplored mechanism to inform future studies and policy – that of *evaluative salience*.

Prior research argues that attention is a constrained resource heavily influenced by context [52–54]. In our study, men on review panels likely focus more on their male peers due to a phenomenon known as *homophily*, the insight that we often pay attention and affiliate with those who look more like us [36–38]. As more women enter the upper ranks and join the review panels, this likely broadens the focus of promotional review to one that is more receptive and fair to female applicants. As evaluators have a direct influence on promotional decisions, we argue that improving salience to gender differences among evaluators is crucial (hence the notion of evaluative salience). And while increasing the gender representation of the applicant pool serves as another mechanism that may alleviate gender discrepancies, we argue an even greater increase in female representation is needed in the applicant pool (compared to the reviewer pool). Perhaps this is because evaluators are a more direct linkage to promotion decisions than are applicants, hence even minimal differences in the gender composition of evaluators arguably have greater impact than the composition of applicants [55].

Such salience-enhancing interventions have already led to significant impacts in other arenas such as entrepreneurship. For instance, showing a stream of pictures of successful projects from a more diverse set of founders can broaden the reach of funding to underrepresented groups [56]. Such subtle interventions are especially important when prior studies note more harm than good when interventions overtly signal gender characteristics [57]. Altogether, we posit that shifts in evaluative salience can help balance expectations between women and men and equalize access to promotional rank. While gender inequality in the sciences persists, evaluative salience offers one mechanism for improving scientific and social progress.

With that in mind, the findings of this study are not conclusive and not without limitations. The study only examines the most productive researchers in terms of publications and citations. For one, there are other research outputs that we do not examine. Moreover, we are limited in our generalizations as we trace the set of elite researchers in Mexico (who select into SNI in the first place). And more work remains on identifying what characteristics foster the phenomenon of greater productivity among women. While there are proportionally fewer women, those women are more productive yet receive fewer promotions.

In conclusion, more work remains on this important issue, and this study is just the onset of this journey towards greater understanding of gender dynamics around promotion in the sciences, especially *outside* the Global North. Within the context of Mexico, future studies should update the timeframe to assess how the trends and mechanisms impacting the administration of SNI have evolved since 2011. Additional work is also needed to examine research activity in other countries outside of the Global Nouth. Comparative studies across different countries or regions could highlight how cultural and institutional context influences gender gaps in research productivity. Investigating how evaluation criteria for promotion or funding decisions will likely help identify biases more precisely and thereby better fine-tune a set of more equitable assessment practices. We hope this direction of scholarship will not only help to generalize our findings, but also develop more scalable recommendations to enhance gender parity in promotion within the sciences.

## Supporting information

S1 File Supplementary tables and figures (S1-S8). (DOCX)
