# Supplementary material for: Unpacking gender discrepancies in academic promotion across STEM fields in Mexico
Source: PLoS One. 2025 Aug 14;20(8):e0324464. doi: 10.1371/journal.pone.0324464 (PMC12352756; doi:10.1371/journal.pone.0324464)
Supplement: S1 File — (DOCX) [file pone.0324464.s001.docx]

**S1 Fig. Dynamics, Female Share to Total.**

*S1 Fig Notes*: Share of female researchers in SNI to total SNI researchers over timeframe of study, 1990 – 2011. Trends designated by promotional rank. Black line reports aggregate trend.

**S2 Fig. Distribution of Rank Over Time.**

**Panel A. Among Females.**

**Panel B. Among Men.**

*S2 Fig Notes*: Panel A reports for women; Panel B reports for men. Each panel reports relative distribution within gender of SNI researchers by promotional rank. Trends reported based on annual estimates over the timeframe of study, 1990 – 2011.

**S3 Fig. Selection Extension.**

**
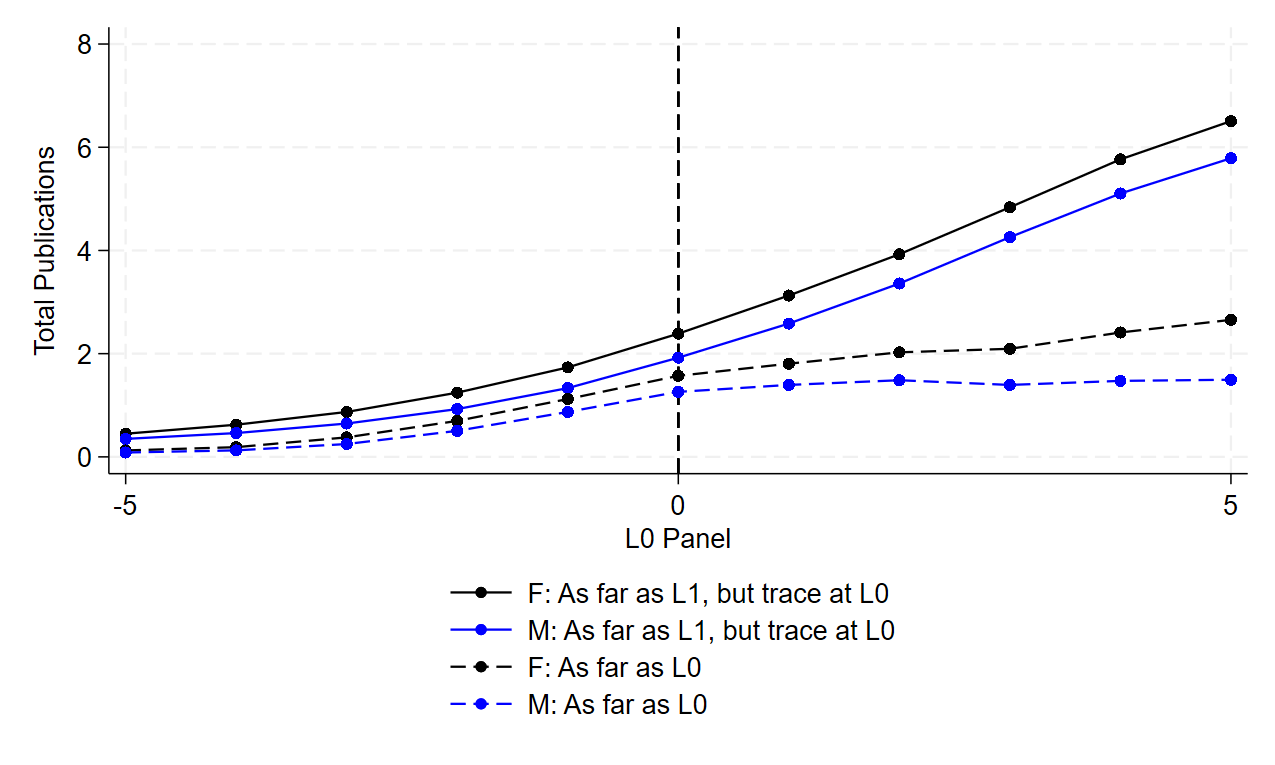
**

**Panel A. Candidate Rank.**

**
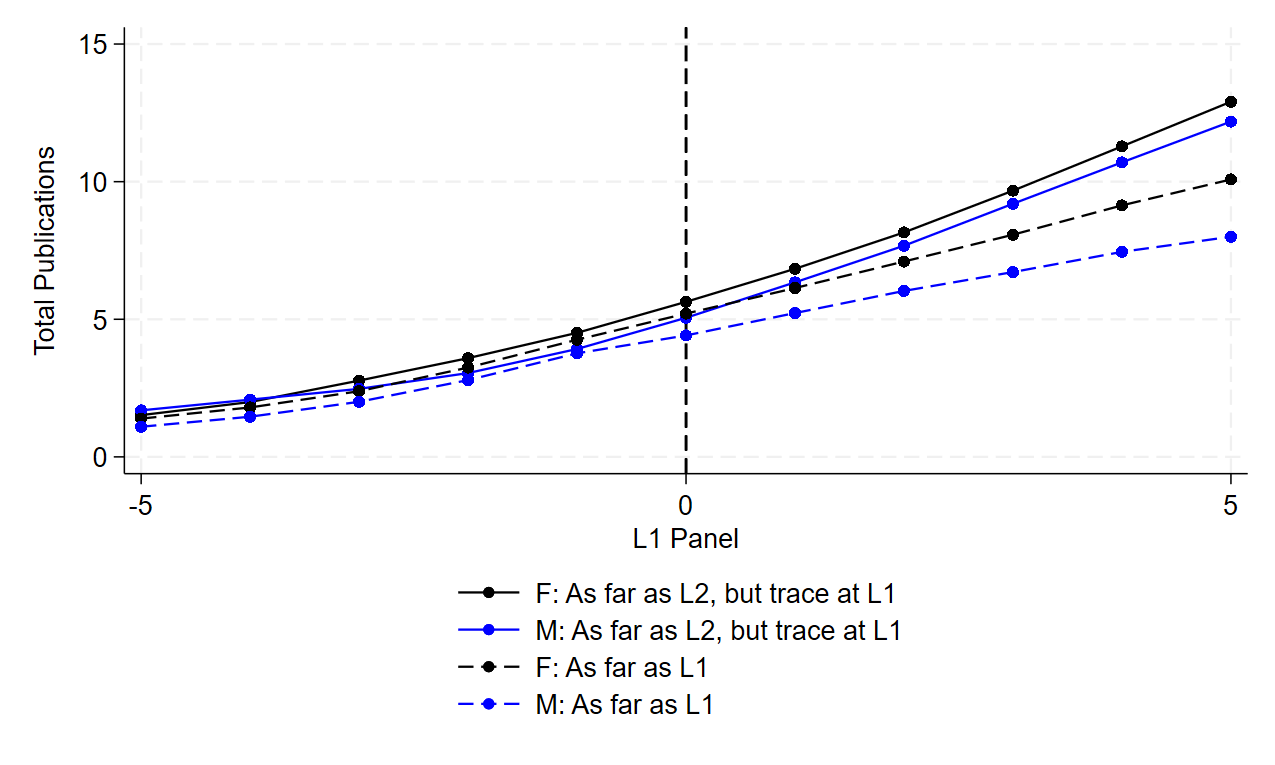
**

**Panel B. Level 1 Rank.**

**
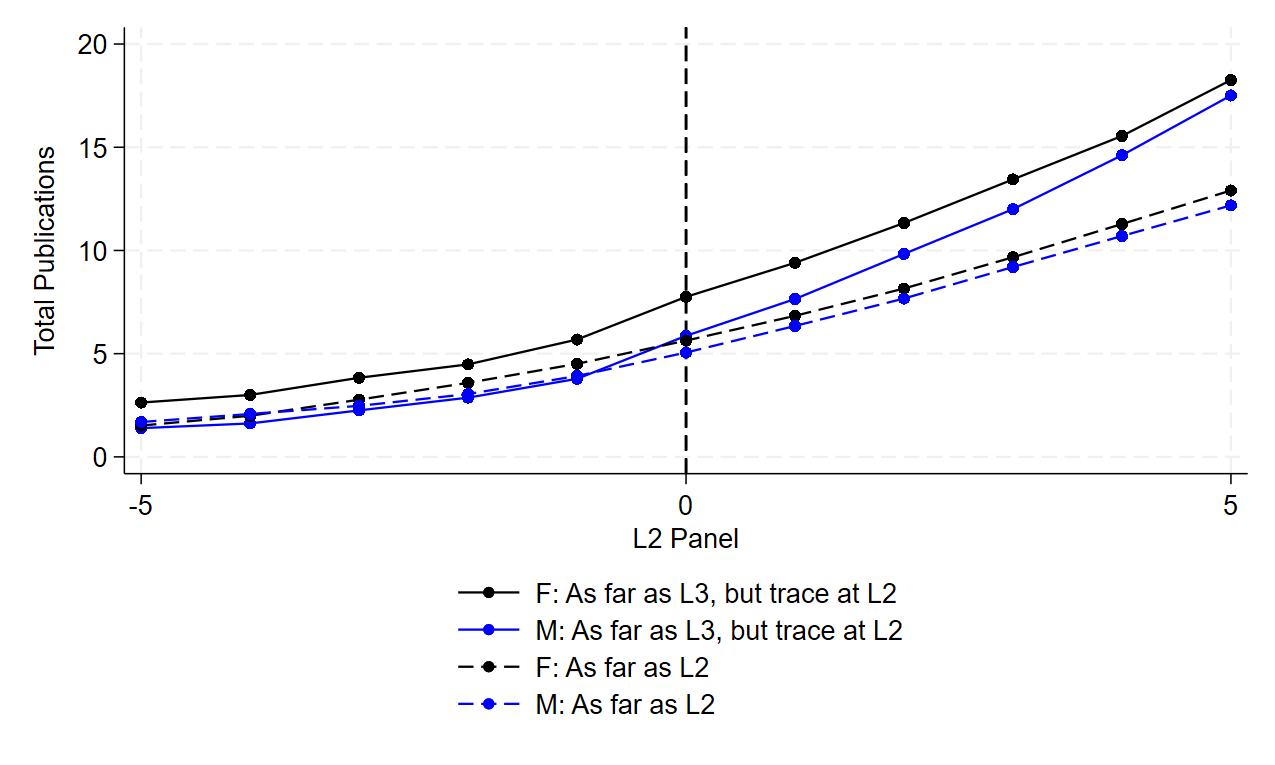
**

**Panel C. Level 2 Rank.**

*S3 Fig Notes*: Image reports aggregate productivity trends using a four-way comparison for each promotional rank. Panel A reports activity around the promotion of Candidate; Panel B reports activity around the promotion of Level 1; and Panel C reports activity around the promotion of Level 2. For each panel, we trace productivity the five years leading up to the promotion (as designated by the panel heading) and the five years following. However, we include trends from both SNI researchers that are only promoted as far as the referent rank (denoted by solid lines) and SNI researchers that ultimately achieve the next higher rank (denoted by dotted lines).

**S4 Fig. History – shifts over timeframe.**

**Panel A. SNI entry before 2001.**

**Panel B. SNI entry on or after 2001.**

*S4 Fig Notes*: Image illustrates dynamic heterogeneity of trends reported in Fig 5. Panel A includes sample of research that entered SNI prior to 2001; Panel B includes sample of researchers that entered on or after 2001. Refer to notes in Fig 5 for detail on formatting.

**S5 Fig. Disciplinary Heterogeneity.**

**Panel A. Physics, Math & Earth Sciences (Area 1).**

**Panel B. Biology & Chemistry (Area 2).**

**Panel C. Medicine & Health Sciences (Area 3).**

**Panel D. Biotechnology & Agricultural Sciences (Area 6).**

**Panel E. Engineering (Area 7).**

*S5 Fig Notes*: Image illustrates heterogeneity of trends reported in Fig 5 by academic area. Refer to panel title for area designation. Refer to notes in Fig 5 for more detail on formatting.

**S6 Fig. PhD Training.**

**Panel A. Mexican PhD.**

**Panel B. Foreign PhD.**

*S6 Fig Notes*: Image illustrates heterogeneity of trends reported in Fig 5 by academic training. Panel A includes SNI researchers that received their PhD from a Mexican institution. Panel B includes SNI researchers that received their PhD from a foreign institution. Refer to notes in Fig 5 for more detail on formatting.

**S7 Fig. Institutional Heterogeneity.**

**Panel A. Large Institution.**

**Panel B. Small Institution.**

*S7 Fig Notes*: Image illustrates institutional heterogeneity of trends reported in Fig 5. Panel A includes SNI researchers with appointments at a large institution. Panel B includes SNI researchers with appointments at a small institution. Refer to notes in Fig 5 for more detail on formatting.

**S8 Table. Productivity by Discipline Within Academic Area.**

| Cumulative Publications | Area Mean | Discipline Min | Discipline Max |
| --- | --- | --- | --- |
| Physics, Math & Earth Sciences – Area 1 (14 disciplines) | | | |
| L0 (Candidate) | 0.68 | 0 | 1.18 |
| L1 | 3.86 | 0.3 | 7.38 |
| L2 | 11.59 | 4.78 | 33.38 |
| L3 | 29.88 | 7.84 | 48.1 |
| Biology & Chemistry – Area 2 (14 disciplines) | | | |
| L0 | 1.15 | 0.05 | 2.9 |
| L1 | 4.47 | 0 | 10.62 |
| L2 | 14.59 | 9.46 | 29.54 |
| L3 | 34.92 | 13.07 | 61.12 |
| Medicine & Health Sciences – Area 3 (14 disciplines) | | | |
| L0 | 1.95 | 0.53 | 6.8 |
| L1 | 6.8 | 0.5 | 12.8 |
| L2 | 19.46 | 4.38 | 24.89 |
| L3 | 52.1 | 34.71 | 74.12 |
| Biotech & Ag Sciences – Area 6 (16 disciplines) | | | |
| L0 | 0.54 | 0 | 2.07 |
| L1 | 2.24 | 0.29 | 3.4 |
| L2 | 7.28 | 2.24 | 17.81 |
| L3 | 18.18 | 8.43 | 41.24 |
| Engineering – Area 7 (25 disciplines) | | |  |
| L0 | 0.57 | 0 | 1.88 |
| L1 | 2.73 | 0.26 | 5.19 |
| L2 | 9.73 | 0.36 | 13.04 |
| L3 | 19.13 | 0.86 | 25.37 |

*S8 Table Notes*: Table reports average cumulative publications by academic area and rank. Column 1 reports mean. The mean accounts for multiple academic disciplines that are part of the SNI area. Col. 2 and 3 illustrate variation within field (i.e., by discipline) and rank reporting minimum and maximum activity.
